# Investigation of Liquid Collagen Ink for Three-Dimensional Printing

**DOI:** 10.3390/mi15040490

**Published:** 2024-04-02

**Authors:** Colten L. Snider, Chris J. Glover, David A. Grant, Sheila A. Grant

**Affiliations:** 1 Arthrex, Naples, FL 34108, USA; colten.l.snider@gmail.com; 2 Pfizer, Chesterfield, MO 63017, USA; christopher.john.glover@gmail.com; 3 Department of Chemical and Biomedical Engineering, University of Missouri, Columbia, MO 65211, USA; grantdav@missouri.edu

**Keywords:** collagen, 3D printing, gold nanoparticles, scaffolds

## Abstract

Three-dimensional printing provides more versatility in the fabrication of scaffold materials for hard and soft tissue replacement, but a critical component is the ink. The ink solution should be biocompatible, stable, and able to maintain scaffold shape, size, and function once printed. This paper describes the development of a collagen ink that remains in a liquid pre-fibrillized state prior to printing. The liquid stability occurs due to the incorporation of ethylenediaminetetraacetic acid (EDTA) during dialysis of the collagen. Collagen inks were 3D-printed using two different printers. The resulting scaffolds were further processed using two different chemical crosslinkers, 1-Ethyl-3-[3-dimethylaminopropyl]carbodiimide hydrochloride)/N-hydroxysuccinimide (EDC/NHS) and genipin; gold nanoparticles were conjugated to the scaffolds. The 3D-printed scaffolds were characterized to determine their extrudability, stability, amount of AuNP conjugated, and overall biocompatibility via cell culture studies using fibroblast cells and stroma cells. The results demonstrated that the liquid collagen ink was amendable to 3D printing and was able to maintain its 3D shape. The scaffolds could be conjugated with gold nanoparticles and demonstrated enhanced biocompatibility. It was concluded that the liquid collagen ink is a good candidate material for the 3D printing of tissue scaffolds.

## 1. Introduction

Collagen is one of the most abundant proteins in the body [1]. This protein provides structural scaffold support for soft tissues such as the dermis, ligaments, and tendons and also plays a role in mechanical protection in articular cartilage [2]. There are 29 different forms of collagen identified [3]. Type I collagen is a highly biocompatible material with low immunogenicity. It can be easily degraded and remodeled naturally by the body’s cells over time, which positions it as an excellent candidate for therapeutic applications and tissue engineering applications [4,5]. Collagen can be derived from many sources including animals such as porcine and bovine [6]. Acquiring type I collagen from a source requires a process of solubilizing various tissue elements until a purified collagen structure can thus be obtained [7,8]. 

Solubilized collagen can be formulated into many types of structures and scaffolds using techniques such as electrospinning, magnetic guidance, and 3D printing via extrusion-based bioprinting [9,10,11,12,13,14,15]. Collagen inks for 3D printing have gained popularity, but these inks not only need to be amendable to extrusion but also maintain their 3D-printed shape after printing [16]. Collagen inks can be printed into solutions with adjusted pH or increased temperatures to initiate collagen fibrilization, forming a solid 3D structure. Additionally, different collagen formulations are possible by mixing with other materials such as alginate, hydrogels, gelatin stem cells, etc. [9,16], prior to 3D printing. Due to the many different formulations of collagen inks, they are usually classified by application as either soft tissue inks or hard tissue inks [16].

Research on the bioprinting of type I collagen has focused on hard tissue applications [17,18] such as the bone, teeth, and spine where the mechanical properties play a key role. Bone regeneration fails once it reaches a critical size defect; thus, 3D printing with collagen inks for hard tissue repair is a growing area of research. A publication by Kim et al. describes the optimization of a type-I-collagen-laden osteoblast-like cell and human-adipose-derived stem cell bioink [19]. In this work, we compared a collagen-based bioink to an alginate bioink. The collagen-based bioink improved cellular activity and also improved relative concentrations of osteogenic biomarkers like BMP-2, Runx2, type I collagen, and OCN at 28 days with human-adipose-derived stem cells [19]. 

Other studies have focused on bioprinting soft tissues [20,21]. Filardo et al. printed an MRI-scanned human meniscus tissue using extrusion-based 3D printing with type I collagen and bone-marrow-derived mesenchymal stem cells [22]. They were able to print an anatomically shaped collagen meniscus scaffold with viable cells. Using additive manufacturing methods like the extrusion-based bioprinting of collagen creates unique fabrication capabilities for patient-specific treatments. 

While 3D-printed collagen inks show promise as scaffolds for tissue recapitulation, they do present some challenges [23]. Pure collagen inks have low mechanical properties, making it difficult for them to maintain the 3D shape after printing. To overcome this limitation, supportive hydrogels may be utilized to act as temporary thermoreversible supports; however, the resulting collagen 3D structures may take 40 to 60 min to fibrillize while also resulting in unwanted supportive hydrogel material trapped within the collagen network. Other collagen inks may require photoinitiators to polymerize the collagen structures [24]. We developed a unique collagen ink that can rapidly fibrillize without any additional initiators or temporary supports to maintain its 3D shape.

Our liquid collagen ink remains in a liquid pre-fibrillized state; liquid stability occurs due to the incorporation of ethylenediaminetetraacetic acid (EDTA) during dialysis of the collagen. Upon interaction with ionic solutions or increased temperatures, the collagen then rapidly fibrillizes [25]. This formulation allows us to load the liquid collagen into syringes where it remains in a stable liquid state for months at room temperature (unpublished data). Our earlier studies have demonstrated the ability of the collagen to undergo fibrillogenesis within 5 min of injection into water or ionic (physiological) solutions at room temperature as well as heated temperatures (37 °C) [25]. Additionally, the extrusion force needed to extrude the collagen through a 30-gauge needle is approximately less than 7 N of force which is much lower than that of commercially available collagen injection products such as SunMax^TM^ (Tainan City, Taiwan), a porcine crosslinked collagen product used in cosmetic anti-aging therapies. Our liquid collagen formulation has mechanical stability upon fibrillization and does not have to be crosslinked during or after the 3D-printing process, which makes it more amendable to different applications, although crosslinking during and after 3D printing is possible. 

To improve the overall biocompatibility properties of collagen scaffolds, we investigated the incorporation of gold nanoparticles (AuNPs) onto the scaffold. AuNPs have had much interest in tissue engineering applications in recent years due to multiple studied benefits such as the mitigation of inflammation, the promotion of cellular migration, and high biocompatibility [26,27,28,29]. For example, we have demonstrated that the attachment of AuNPs to decellularized porcine diaphragm tissue can enhance the lifetime of the scaffold; the AuNPs can hinder collagenase binding sites and thus extend the lifetime of the scaffold [29]. We have shown that the presence of AuNPs on implanted decellularized porcine tissue can reduce inflammation [30,31]. Additionally, the attachment of AuNPs to scaffolds may increase the surface energy of the scaffold which can in turn increase cellular adherence through the adsorption of proteins [32]. AuNPs have also been documented to be an effective antimicrobial agent along with being an effective free radical scavenger which inhibits the formation of reactive oxygen species (ROS) [33,34]. The production of ROS is known to be detrimental to tissues during wound healing. Utilizing AuNPs on musculoskeletal tissue scaffolds may allow for quicker healing time through increased cellular migration, remodeling, and reduction of surrounding ROS. In this study, we investigated the feasibility of conjugating AuNPs to 3D-printed collagen structures via crosslinkers. 

In our study, the utilization of chemical crosslinkers to attach AuNPs is critical. The crosslinker should improve the overall mechanical properties, but it should not be detrimental to the overall biocompatibility. Commonly used chemical crosslinkers include genipin and 1-Ethyl-3-[3-dimethylaminopropyl]carbodiimide hydrochloride)/N-hydroxysuccinimide (EDC/NHS). EDC/NHS crosslinking involves the formation of a peptide bond between carboxyl and amino groups. An advantage of the EDC/NHS crosslinker is that it is a zero-length crosslinker, meaning the actual EDC molecule is not a part of the final crosslinked product. A disadvantage is the EDC/NHS crosslinking reaction creates unwanted urea byproducts that can cause cytotoxicity if not removed. This then requires laborious washing techniques, which are time-intensive and may damage the 3D collagen scaffolds. EDC/NHS crosslinking has also been shown to affect native-like cellular adhesion [35]. An alternative crosslinker is genipin. Genipin is a natural crosslinker isolated from gardenia jasminoides fruits. Genipin can spontaneously react with two amino groups that form monomer-to-tetramer crosslinks [36]. The use of genipin has also been studied as an anti-inflammatory agent [37,38]. Genipin is generally advantageous because it does not necessitate extensive washing steps to remove extraneous byproducts after the crosslinking process. 

This work describes the method of fabricating type I collagen scaffolds for general soft tissue applications via additive manufacturing and 3D-printing techniques. We are one of the first groups to report on utilizing EDTA-stabilized collagen as 3D-printing inks. Collagen inks were 3D-printed using two different printers. The resulting scaffolds were further processed using two different chemical crosslinkers, EDC/NHS and genipin; AuNPs were conjugated to some of the scaffolds and characterized. The 3D-printed scaffolds were characterized to determine their extrudability, stability, amount of AuNP conjugated, and overall biocompatibility via cell culture studies using fibroblast cells and stroma cells. 

## 2. Materials and Methods

### 2.1. Fabrication of Liquid Collagen

Porcine collagen type I (6 mg/mL, Sunmax Biotechnology, Tainan City, Taiwan) was utilized as our collagen ink. It was precipitated using 1.04 M sodium chloride (NaCl, ≥99.0%, Sigma Aldrich, St. Louis, MO, USA). The precipitated collagen solution was then centrifuged at 3500 rpm for 15 min. Once a white collagen pellet was formed at the bottom of the tube, the supernatant was poured off, leaving a 150 mg collagen pellet. A total of 15 mL of 0.5 M glacial acetic acid (≥99.7%, Fisher Chemical, Lenexa, KS, USA) was added to the collagen pellet. To let the collagen pellet solubilize, it was allowed to sit overnight at room temperature. The collagen/acetic acid solution was then placed in a 15 mL, 10 kDa molecular weight cutoff dialysis cassette (Thermo Scientific, Bannockburn, IL, USA) and immersed in ethylenediaminetetraacetic acid (35 mM, EDTA, Fisher Chemical, Lenexa, KS, USA)/H_2_O solution with a pH of 7.5 using sodium hydroxide (10 N, NaOH, Ricca Chemical Co., Arlington, TX, USA) [17]. The pH of the EDTA solution was monitored and maintained at 7.5 daily until the pH no longer fluctuated from 7.5. The liquid collagen (LC) solution was then removed, and pH was tested to ensure a pH of 7.5. The resulting collagen concentration was ~25 mg/mL.

### 2.2. Bioprinters

**Figure 1:**
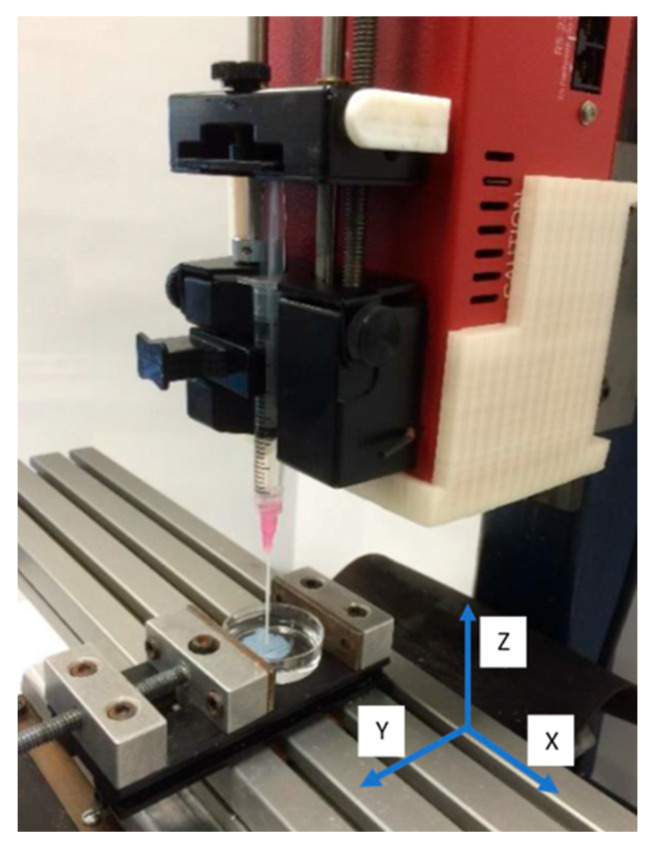
Custom 3D printer from CNC milling machine. *X*, *Y*, and *Z* axes are denoted on printer. The image shows 3D printer printing collagen scaffold.

**Figure 2:**
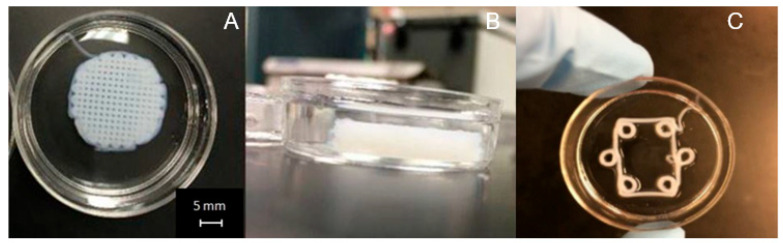
Collagen scaffolds printed from custom 3D printer: (**A**) top view of 20 mm × 6.3 mm 3D-printed collagen cylinder scaffold; (**B**) side view of 20 mm × 6.3 mm 3D-printed collagen cylinder scaffold; (**C**) 4 mm × 2.4 mm 3D-printed cylinder scaffolds.

Two different printers were utilized. One of the printers used to fabricate the 3D scaffolds was a custom printer assembled from a CNC milling machine, as shown in Figure 1. Three stepper motors were used to individually control each axis: X, Y, and Z. The motors had an error of movement less than 0.1 μm. The print bed was created by placing the X stage and Y stage on top of one another. The Z stage was placed in a perpendicular orientation to the print bed. To control the motor’s movements, G-code was written and executed using Mach3 Mill software (3.043.062 version). A syringe pump was mounted on the Z stage which held and extruded the printing solution (liquid collagen). Figure 2 shows images of scaffolds printed from the custom CNC milling machine. Figure 2A,B are images of a 20 mm × 6.3 mm cylinder printed into a solution of 15 mL of 24 °C ddH_2_O. Post printing, scaffolds were allowed to fibrillize for 5 min and then lifted out of the Petri dish and placed in 70% ethanol for storage.

**Figure 3:**
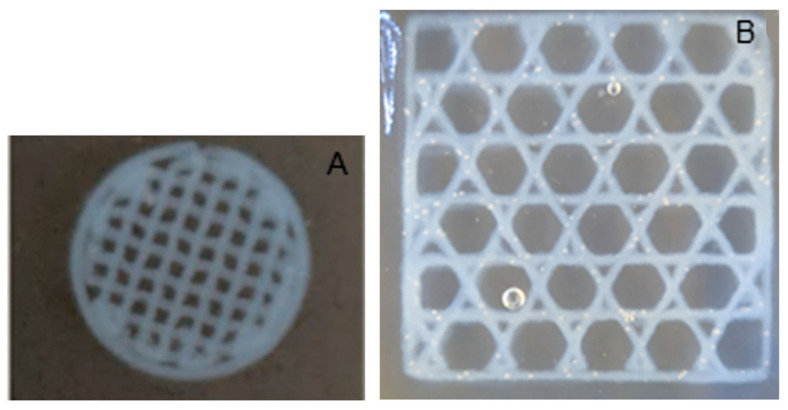
Three-dimensionally printed collagen scaffolds using Cellink BioX printer: (**A**) 6 mm × 0.8 mm cylinder scaffold printed in water; (**B**) 20 mm × 20 mm × 1 mm rectangular scaffold printed in agarose microparticle solution.

A Cellink Bio X (Boston, MA, USA) 3D bioprinter using a temperature-controlled pneumatic printhead (4–65 °C) was also utilized to print the scaffolds, as shown in Figure 3. A 3 mL Cellink pneumatic printing cartridge was filled with our printing solution (liquid collagen), and a 27-gauge conical nozzle was utilized during printing. The temperature-controlled pneumatic printhead was set to 4 °C. The printing solution was printed into a 150 mm diameter polystyrene Petri dish filled with 15 mL of 24 °C ddH_2_O. The print height was set to 0.2 mm with a printing speed of 3 mm/s, and nozzle pressure was set to 10 kPa for all prints. With the Cellink bioprinter, we also examined the resulting 3D-printed collagen fiber diameters under various extrusion pressures using a 27-gauge nozzle at 3 mm/s. Post printing, scaffolds were allowed to fibrillize for 5 min and then lifted out of the Petri dish and placed in 70% ethanol for storage. Three-dimensional models were designed using Solidworks (standard, 2018)and sliced with Cellink’s built-in slicing software or Slic3r software (2017, standard). 

We also investigated printing the collagen ink in an agarose microparticle solution, a crosslinking solution, and cell culture media suspension with L929 fibroblast cells. The agarose microparticle printing solution was developed by Senior et al. [39]. To prepare the solution, a 0.5% (*w*/*v*) agarose solution was heated in an autoclave for 30 min at 225 °F. After autoclaving, the heated solution was placed on a stir plate at 700 rpm until the solution reached room temperature. 

Figure 3 shows the 3D-printed collagen scaffolds using the Cellink BioX printer. Figure 3A is a 6 mm × 0.8 mm cylinder scaffold (4 layers) with a 20% rectilinear infill pattern that was printed in water. Figure 3B is a 20 mm × 20 mm × 1 mm rectangular scaffold printed in an agarose microparticle solution. For cell studies, the scaffold design in Figure 3B was utilized.

### 2.3. EDC/NHS Crosslinking

To crosslink the 3D-printed scaffolds using EDC/N-hydroxysulfosuccinimide (NHS) crosslinking, printed scaffolds were placed in a flask at room temperature in a solution of 2 mM EDC (dissolved in 0.1 M 2-(N-morpholino)ethanesulfonic acid (MES) in 0.5 M sodium chloride (NaCl)) and 5 mM sulfo-NHS (first dissolved in dimethylformamide (DMF)) 50% acetone and phosphate-buffered saline (PBS; 7.5 pH). The flasks were placed on an orbital shaker at 75 rpm for 12 h. Samples were subsequently washed five times with PBS. We also tested printing the collagen ink directly into the EDC/NHS crosslinking solution and compared its thermal characteristics. 

### 2.4. Genipin Crosslinking

To crosslink the genipin-crosslinked scaffolds, 2 mM genipin (initially dissolved in 18% (*w*/*v*) dimethylsulfoxide (DMSO)) solution was prepared using PBS. Samples were placed in a flask on an orbital shaker at 75 rpm and were incubated in the genipin solution for 12 h. Samples were subsequently washed three times with PBS.

### 2.5. Conjugation of AuNPs

AuNPs with the size of 20 nm were purchased from Ted Pella Inc. (Redding, CA, USA) at a concentration of 7.0 × 10^11^ AuNP/mL, which correlates to a 1x AuNP concentration. The size (9.0–21.0 nm) and concentration are guaranteed by Ted Pella, Inc., and we have previously utilized and examined the size [29]. To conjugate AuNPs, AuNPs were functionalized using a 15 µM 2-mercaptoethylamine (MEA) solution and added to the printed scaffolds at the same time as the addition of 2mM EDC/NHS crosslinking solution or 2 mM genipin crosslinking solution [40]. A 2× concentration correlates to a 14.0 × 10^11^ AuNP/mL concentration of AuNPs, which was achieved by spinning down the AuNP and siphoning off half the solution. A 1× AuNP concentration was utilized unless otherwise stated.

### 2.6. Sterilization 

Samples undergoing biological study were sterilized using an ethanol solution. For 24 h at room temperature, samples were placed in a 70% ethanol solution. After 24 h, samples were then placed in sterile cell media for 24 h. Finally, samples were transferred to a sterile 48-well culture and immersed in fresh sterile cell media in preparation for biological culture.

### 2.7. Differential Scanning Calorimetry

To compare the denaturation temperatures of the 3D-printed scaffolds to determine their stability, a Q2000 Differential scanning calorimeter (DSC) (TA Instruments, New Castle, DE, USA) was utilized. The 3D-printed scaffolds were printed, dissected, and then placed in the bottom of aluminum sample pans (~5 mm in diameter). These pans were then hermetically sealed. The DSC heated from −5 °C to 120 °C with a temperature ramp rate of 3 °C/min with modulation every 80 s ± 0.64 °C. The denaturation temperatures were determined using Universal Analysis software (Standard, 2007).

### 2.8. Scanning Electron Microscopy

Scanning electron microscopy (SEM) was used to determine AuNP conjugation to 3D-printed scaffolds. Images were acquired using a Quanta 600 FEG (FEI, Hillsboro, OR, USA). Magnification ranged from 75× to 20,000×, and the electron beam was set to 10 kV. Samples were in low vacuum.

### 2.9. Neutron Activation Analysis

To quantify the gold nanoparticles bound to the scaffolds, neutron activation analysis (NAA) was utilized. NAA was performed at the University of Missouri Research Reactor (MURR). Once printed, the collagen samples were lyophilized, weighed, and secured within high-density polyethylene vials where they remained during the analysis. Samples were irradiated for two minutes and then allowed to decay for one to seven hours. Gamma radiation was measured for ten minutes via a Canberra High Purity Germanium detector. The detector has a relative efficiency of 33.7% and a full-width half-maximum resolution of 1.73 keV at 1.33 MeV. A Canberra digital signal processor, Model 9660A, was used in tandem with the detector and a high-voltage power supply. Analysis of the data was performed utilizing Canberra-VMS Genie 2000 software, and the quantities of gold were recorded.

### 2.10. Cell Culture Studies 

Cell assays were conducted with L929 murine fibroblast cells acquired from ATCC Manassas, VA, to assess biocompatibility. Cells were cultured at 37 °C and 5% CO_2_. Cell media used for culture was Eagle’s Minimum Essential Medium (EMEM) supplemented with 200 U/mL Penicillin streptomycin and 10% horse serum. Cells remained under sterile conditions using a biological safety cabinet. Cell passage numbers in the assays were between two and twenty-eight times. Cell viability reagent WST-1 (Sigma Aldrich, MO, USA) was used to assess the biocompatibility of the 3D-printed scaffolds with L929 murine fibroblast cells. The 3D-printed scaffolds were incubated in fresh supplemented EMEM 24 h prior to the addition of fibroblast cells in a 48-well plate. Cells were seeded onto scaffolds at a ratio of 3 × 10^4^ cells per well. A total of 250 µL of the supplemented media was replaced every 3 days during study. A total of 50 µL of the WST-1 reagent was added to each well and allowed to incubate for 4 h. After 4 h, 125 µL from each well was plated into a new 48-well plate, and absorbance readings were measured at 450 nm with a 600 nm filter using a spectrofluorometer. 

**Figure 4:**
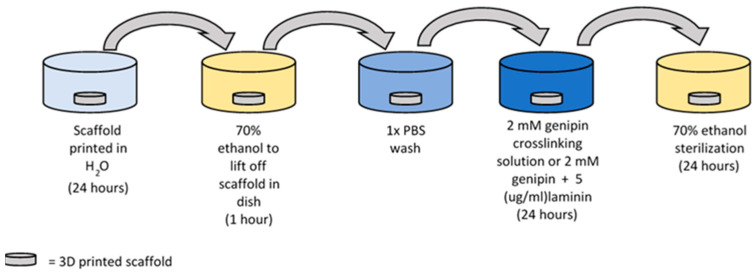
Diagram of the 3D-printed collagen scaffolds prepared for culture with stromal cells.

Additionally, the ability of the 3D scaffolds to host and support stromal cells was assessed via visual microscopy. Stromal cells were provided by Harriet Fitzgerald from the University of Missouri Animal Science division. The cells were seeded onto modified 3D-printed collagen scaffolds that were printed in water using the Cellink bioprinter. Three separate groups were studied. In the first group, genipin (2 mM) was used to crosslink the 3D-printed collagen fibers to create a more stable structure. In the second group, laminin, a basement membrane protein (5 μg/mL), was added to the scaffolds in order to coat the scaffolds and thus create improved cellular adherence to the 3D-printed scaffolds. The third group was a combination of scaffolds crosslinked in genipin and laminin. Figure 4 provides a flow chart on how these scaffolds were prepared for culture.

### 2.11. Statistical Analysis

Unless stated otherwise, data represent the mean of three independent replicates, and error bars represent the standard error of the mean. Each independent replicate was conducted in triplicate for each sample. Statistical comparisons were performed using one-sided or two-sided *p*-values, which were calculated using one-way analysis of variance. A *p*-value less than 0.05 was considered to be statistically significant.

## 3. Results

### 3.1. Three-Dimensionally Printed Scaffold Characterization 

Figure 2 provides images of 3D-printed collagen scaffolds using the liquid collagen (LC) solution on the custom CNC bioprinting machine. As shown in Figure 2A,B, a general 20 mm × 6.3 mm cylinder was printed in order to determine if the LC was amendable to 3D printing. The images demonstrated that the LC is amendable to printing into a solution resulting in fibrillization. The scaffold maintained its shape and structure, even with a thickness of over 6 mm. The average diameter of the collagen strands was 500 μm. The 3D-printed scaffolds observed in Figure 2C were used in our characterization studies. 

**Figure 5:**
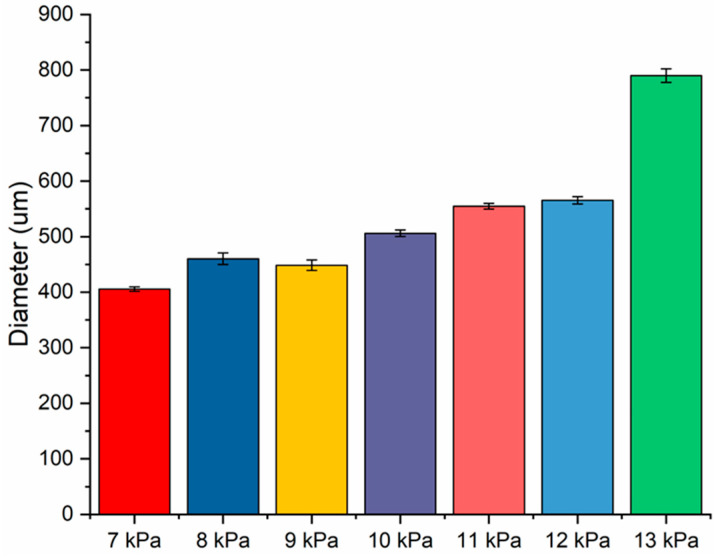
Three-dimensionally printed collagen fiber diameter of various extrusion pressures from Cellink BioX printer using 27-gauge nozzle at 3 mm/s.

Samples produced using the Cellink BioX printer can be seen in Figure 3. The 3D-printed scaffold in Figure 3A was printed into a water solution and was used in our cellular characterization studies. The 3D-printed scaffold in Figure 3B was printed using the support agarose solution which provides solid-like behavior under low shear but liquid-like behavior with applied stress [39]. The support solution is also able to recover from the deformation quickly once the shear stress is removed. We utilized the support agarose in order to determine if we could obtain a more controlled structure and shape of the collagen scaffold. The average diameter of the collagen strands was dependent upon extrusion pressure. Using a 27-gauge conical bioprinting nozzle tip from Cellink, a profile of LC’s print diameter was determined by holding the printing rate a 3 mm/s. The results are shown in Figure 5. Printed diameters ranged from 400 μm to 802 μm. Noticeably, an increase in printed diameter was observed with increased extrusion pressure ranging from 7 kPa to 13 kPa. A significant increase in printed diameter was observed from 12 kPa to 13 kPa, which demonstrated an approximately 224 μm increase in diameter. Printing below 6 kPa was not possible due to the difficulty of extruding collagen under low pressures while printing above 13 kPa resulted in non-uniform collagen diameters.

### 3.2. Thermal Stability

**Figure 6:**
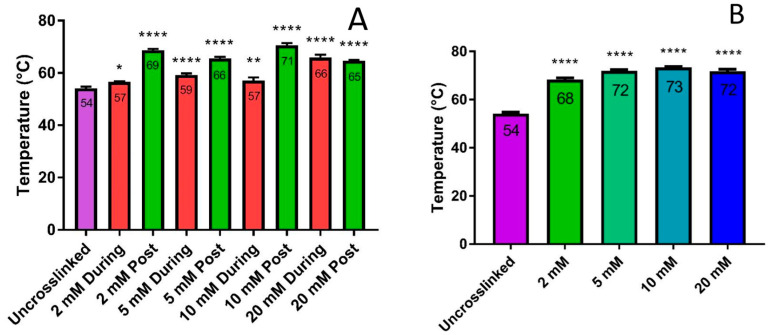
DSC denaturation results. (**A**) Crosslinking during the printing process where collagen was fibrillized in the EDC/NHS solution and crosslinking after collagen fibrillization in post printing; (**B**) crosslinking using genipin after collagen fibrillization in post printing. (* *p*-value < 0.05; ** *p*-value < 0.01; **** *p*-value < 0.0001).

The denaturation temperatures of samples printed in both EDC/NHS and genipin were analyzed. In our preliminary experiments with EDC/NHS crosslinking, two methods of crosslinking were tested. In one method, the samples were printed directly into the crosslinking solution and stayed in the solution for 24 h. In the second method, the samples were printed into water, stayed in water for 24 h, and were then placed in the crosslinking solution. The denaturation results are shown in Figure 6A. These results demonstrated that both methods increased the overall denaturation temperature relative to the uncrosslinked sample. Interestingly, the two crosslinking techniques demonstrated significantly different results. Crosslinking in 2 mM, 5 mM, and 10 mM, the samples that were printed in water first and then crosslinked had a much higher denaturation temperature compared to the samples that were printed directly into the crosslinking solution. Alternatively, the group that was printed directly into the crosslinking solution had a higher denaturation temperature in comparison to printing in water then crosslinking when utilizing the higher molar concentration of EDS (at 20 mM). Overall, the 10 mM EDC crosslinking group that was printed into water then crosslinked had the highest thermal stability at 71 °C. The 2 mM EDC/NHS was utilized in future studies unless otherwise stated. Using a 2 mM EDC/NHS crosslinker provided the structural stability of the collagen scaffold without the rigidity. If the scaffold is too stiff, then the collagen’s ability to achieve enhanced cellularity could be adversely impacted. The 2 mM solution provided an increased denaturation temperature and helped to maintain the printed dimensions, which allowed the handling of the scaffolds. 

Samples crosslinked with genipin were all printed into water and then placed in a genipin crosslinking solution. All samples crosslinked with genipin were significantly more stable than uncrosslinked samples, as shown in Figure 6B. An increase in denaturation temperature can be observed by increasing the genipin crosslinking solution from 2 mM to 10 mM. The increased denaturation temperature from 2 mM to 5 mM was the most significant with an overall decrease in denaturation temperature observed from 10 mM to 20 mM. With 2 mM genipin providing a significant increase in thermal stability, 2 mM genipin was used in all future studies unless otherwise stated. 

### 3.3. SEM Analysis

**Figure 7:**
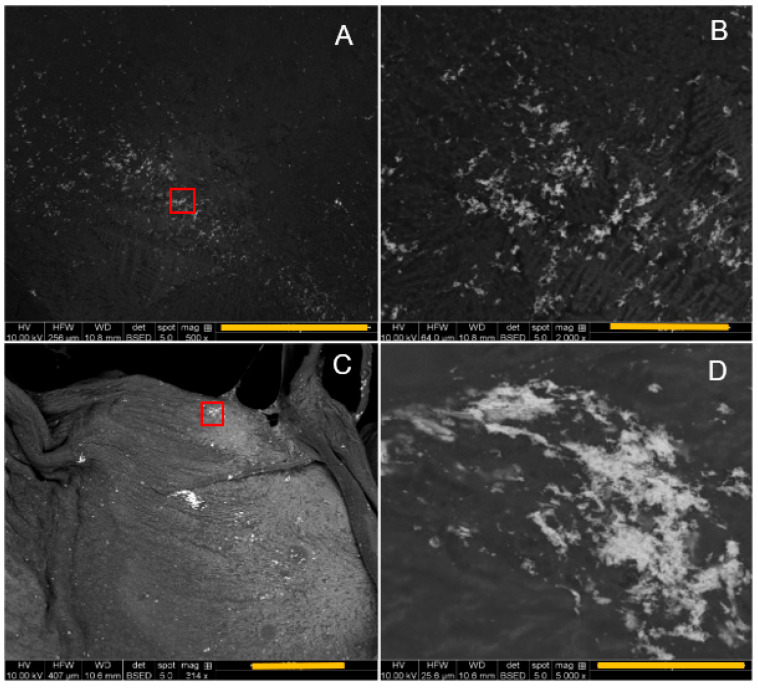
SEM backscattered micrographs of EDC/NHS conjugated AuNPs on 3D-printed scaffolds: (**A**) 500× magnification; (**B**) 2000× magnification from red box in (**A**); (**C**) 314× magnification; (**D**) 5000× magnification from red box in (**C**). Scale bar = 100 mm in (**A**,**C**); 20 mm in (**B**); 10 mm in (**D**).

**Figure 8:**
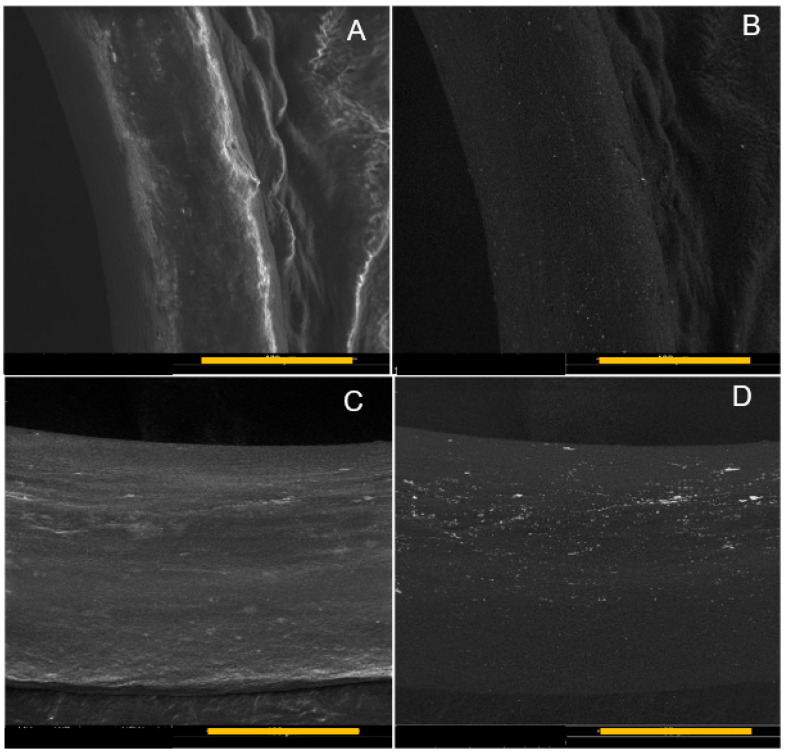
SEM micrographs of genipin-conjugated AuNPs on 3D-printed scaffolds: (**A**) secondary electron micrograph, 1× AuNP concentration; (**B**) backscattered electron micrograph, 1× AuNP concentration; (**C**) secondary electron micrograph, 2× AuNP concentration; (**D**) backscattered electron micrograph, 2× AuNP concentration. Scale bar is 100 μm.

Both EDC/NHS and genipin crosslinking was used to conjugate AuNPs to the 3D-printed collagen scaffolds, which were analyzed using SEM microscopy to validate the presence of AuNPs, as shown in Figure 7 and Figure 8, respectively. Both crosslinking techniques had AuNPs conjugated to the surface of the printed collagen fibers. Upon visual inspection, the samples conjugated with EDC/NHS appeared to have more clumped AuNPs on the surface of the scaffolds relative to the samples conjugated with genipin.

### 3.4. NAA Analysis

**Figure 9:**
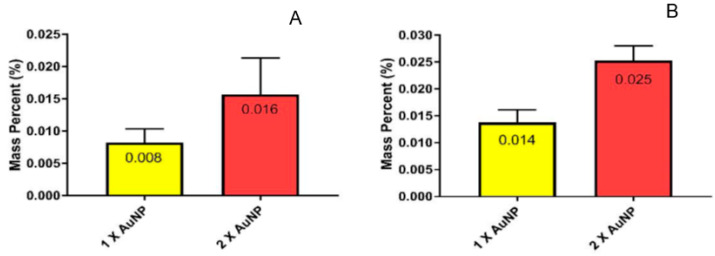
NAA results from crosslinking AuNPs to 3D-printed scaffolds with (**A**) EDC/NHS and (**B**) genipin.

NAA analysis was used to provide a quantitative measurement of relative AuNPs conjugated to the 3D-printed scaffolds. The results of the conjugation of 1× and 2× concentrations of AuNPs with both EDC/NHS and genipin using 2 mM concentrations for both types of crosslinkers are shown in Figure 9. On average, when comparing both cases of 1× and 2× AuNP concentrations, genipin demonstrated more efficiency when conjugating as compared to EDC/NHS conjugation in terms of mass percent. Comparing the increase from 1× to 2× AuNP, both genipin and EDC/NHS demonstrated increases in conjugation. EDC/NHS demonstrated an approximately 100% increase in AuNP mass percent, as shown in Figure 9A (0.008 to 0.016). Genipin demonstrated an approximately 78% increase in AuNP attachment, as shown in Figure 9B (0.014 to 0.025).

### 3.5. Three-Dimensional Printing into A Cell Suspension

**Figure 10:**
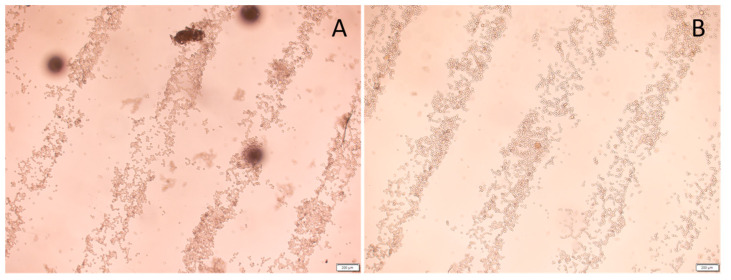
Printing collagen scaffold into L929 cell solution: (**A**) 4× light microscope image of cells on collagen fibrils immediately after printing without washing; (**B**) 4× light microscope image of cell on collagen fibrils after 10 min of incubation and 5 washes. Scale bar is 200 μm.

The printing of a 3D collagen scaffold into an L929 fibroblast cell solution was investigated using light microscopy. Figure 10 provides images of a 3D-printed scaffold printed into 4 mL of a 4.0 × 10^5^ cells/mL L929 fibroblast solution. The goal of this study was to determine if printing into a cell solution with a 10 min incubation would provide enough time for the cells to adhere to the 3D scaffold. Figure 10A is an image immediately after the printing of the scaffold. The outline of the collagen fibers can be observed due to the cell covering adhering to the fibers. After a 10 min incubation of the cells at 37 °C, the 3D scaffold was washed five times using cell media and imaged again, as shown in Figure 10B. After washing the scaffold, a significant number of cells remained on the 3D-printed scaffold. 

### 3.6. Cell Viability Analysis

**Figure 11:**
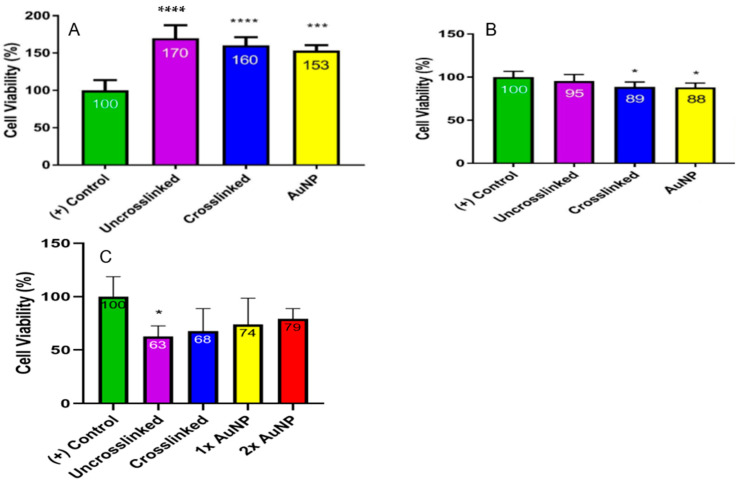
WST-1 cell viability analysis with L929 murine fibroblast cells: (**A**) 3 days of culture with genipin crosslinked; (**B**) 3 days of culture with EDC/NHS crosslinked; (**C**) 7 days of culture with genipin crosslinked. (* *p*-value < 0.05; *** *p*-value < 0.001; **** *p*-value < 0.0001).

A 3-day cell viability analysis was conducted using both EDC/NHS and genipin crosslinkers with AuNPs. The results are shown in Figure 11. All groups with 3D-printed collagen scaffolds had enhanced cellular viability compared to cells with no scaffold, as shown in Figure 11A. A slight reduction in viability can be observed between the uncrosslinked collagen scaffolds compared to the genipin-crosslinked scaffolds and also the genipin scaffolds conjugated with AuNPs. Using EDC/NHS crosslinking, similar cellular viability can be observed between groups with 3D-printed collagen scaffolds and cells with no scaffolds, as shown in Figure 11B. Similar to the use of genipin and the addition of AuNPs, EDC/NHS crosslinking along with the addition of AuNPs decreased the cellular viability relative to cells with no scaffold. 

A 7-day cell viability analysis was also conducted looking at the use of crosslinker genipin and 1× and 2× AuNP concentrations (Figure 11C). Cells with no scaffold had increased viability relative to cells with 3D-printed collagen scaffolds. The addition of genipin to the scaffolds slightly increased the viability relative to the uncrosslinked scaffolds. The conjugation of 1× and 2× AuNP further enhanced viability relative to genipin alone, with 1× AuNP providing the highest overall viability relative to all groups with 3D-printed scaffolds. 

### 3.7. Printing in Agarose Solution

**Figure 12:**
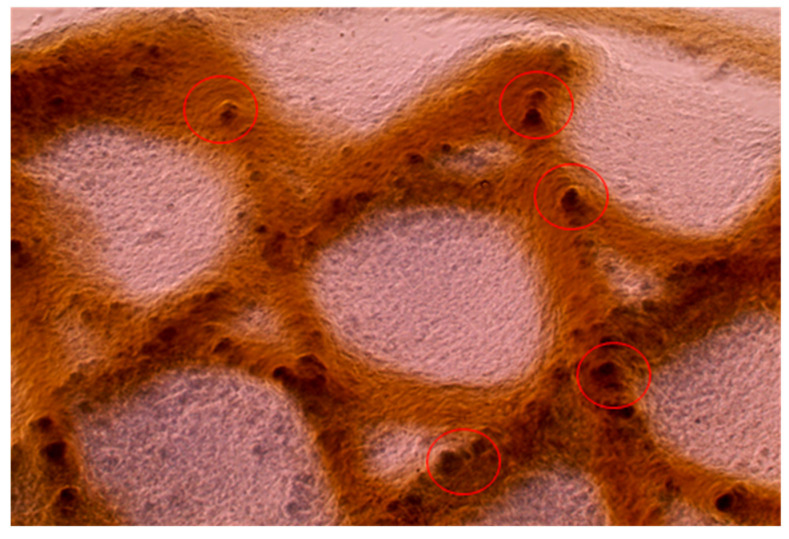
A 4× light microscope image of a 3D-printed collagen scaffold printed into an agarose microparticle solution. The scaffold was washed prior to imaging. Red circles highlight some of the remnant agarose particles on the scaffold. The collagen structure has approximately 500 μm in diameter fibers.

Printing into an agarose microparticle solution was attempted as shown earlier in Figure 3B and cultured with cells. Printing into the agarose microparticle solution was advantageous due to the almost 100% reproducibility of each scaffold. However, concerns were noted when preparing the 3D-printing scaffolds for cell studies. The first complication was washing the scaffolds after printing. The agarose needed to be removed from the scaffold post-printing. After washing, remnant agarose microparticles were still apparent on the 3D-printed scaffold, as shown in Figure 12. Secondly, cells seeded onto the scaffold did not appear to have much if any attachment on the scaffold, preferring the bottom of the well plate instead. 

### 3.8. Stromal Cell Degradation of 3D-Printed Scaffolds

**Figure 13:**
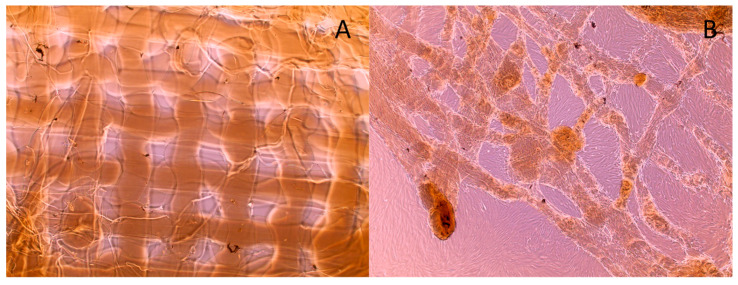
The 4× light microscope images of 9-day cultured 3D-printed collagen scaffolds with no crosslinker: (**A**) with no cells; (**B**) with stromal cells. The collagen structure has approximately 500 μm in diameter fibers.

Stromal cells were seeded onto a 10 mm × 0.6 mm uncrosslinked 3D-printed collagen scaffold that was printed in water. Figure 13 provides images of the scaffold at 9 days of incubation without cells (Figure 13A) and with the stromal cells (Figure 13B). The cells appeared to significantly degrade the 3D-printed scaffold at 9 days relative to the sample with no cells. The cells also began to seed along the bottom of the well plate. 

**Figure 14:**
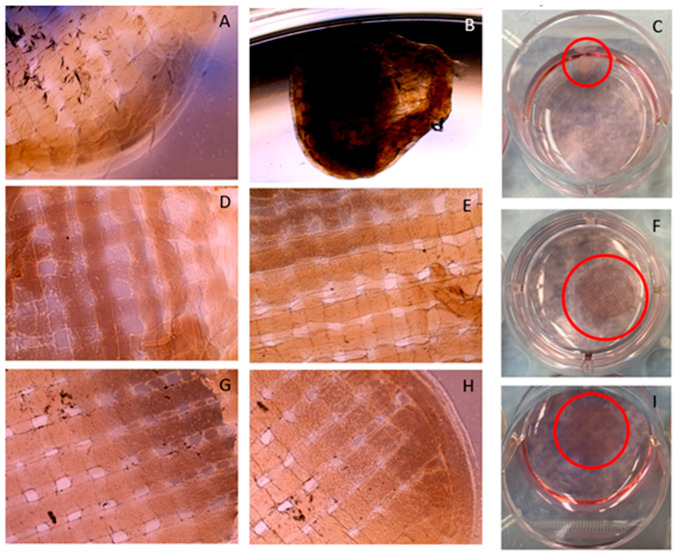
Images of stromal cells seeded onto 3D-printed collagen scaffolds supplemented with (**A**–**C**) laminin; (**D**–**F**) genipin crosslinked; (**G**–**I**) genipin crosslinked and laminin. (**A**,**D**,**G**) are 4× light microscope images at 4 days of incubation; (**B**,**E**,**H**) 4× light microscope images at 16 days of incubation; (**C**,**F**,**I**) photographs at 16 days of incubation. The collagen structure has approximately 500 μm in diameter fibers. The red circles indicate the location of the 3D printed scaffolds.

This study was repeated with different 3D-printed scaffolds and stromal cells. Three separate groups were studied: genipin scaffolds, laminin scaffolds, and genipin + laminin scaffolds. Laminin, a basement membrane protein, was added to the scaffolds in an attempt to improve cellular adherence to the 3D-printed scaffolds. The results of the scaffolds seeded with stromal cells can be observed in Figure 14. Images were acquired at 4 days and 16 days of culture. On day 4 of culture, the scaffold with only laminin began shrinking, and visually the pores of the scaffold were lost, as noted in Figure 14A. Both samples crosslinked with genipin remained structurally sound, and their shape was retained, as shown in Figure 14D,G. Cells can be observed on the surface of both genipin-crosslinked scaffolds at 4 days. At 16 days, the laminin-only sample had completely lost its structural integrity and formed a globular shape, as noted in Figure 14B,C. The two genipin-crosslinked samples remained structurally intact at 16 days, as shown in Figure 14E,H. Again, both genipin-crosslinked samples retained a homogenous cellular network across their scaffolds. Figure 14C,F,I are photographs of the scaffolds at 16 days. The scaffolds’ porous network is still visible by the naked eye with samples crosslinked with genipin while the uncrosslinked, laminin sample demonstrated a visible reduction in size and a loss of integrity. 

## 4. Discussion

Three-dimensional printing provides more versatility in the fabrication of scaffold materials for hard and soft tissue replacement, but a critical component is the ink. The ink solution should be stable over time to allow for versatility of use. The ink should be amendable to be printed into different solutions to allow for the incorporation of crosslinking agents, cells, peptides, etc. Once printed, the scaffolds need to maintain their shape, size, and function [16,41]. Biocompatibility is also a critical requirement before, during, and after printing. In this work, we investigated the potential to 3D print a pre-fibrillized, liquid collagen formulation for its potential as an ink. 

Our collagen ink solution is a clear, transparent liquid in a pre-fibrillized state at a neutral pH. We have worked extensively with this liquid collagen as an injectable biomaterial for various soft tissue replacements [25,42]. Our previous work demonstrated that the liquid can be stable at room temperature for months without fibrillizing. It was also determined that this inhibition of fibrillogenesis may be due to the presence of EDTA which surrounds the concentrated solution of the triple helical collagen molecules. Additionally, the inhibition of the fibrillogenesis may be due to ionic interactions occurring at several different regions of collagen molecules [25]. Upon interaction with solutions, it is assumed that most of the EDTA and/or ions are displaced, mitigating the shielding and thus allowing fibrillogenesis to occur. The displacement of EDTA into its surroundings would have additional benefits. EDTA has been shown to be an antimicrobial and antibiofilm agent [43] and thus could reduce the chances of infections in implanted scaffolds. The time response to initiate fibrillogenesis has been examined extensively [25]; fibrillization is initiated immediately, and complete fibrillization depends on the size of the bolus injected but usually occurs within minutes. Additionally, we have performed an in vivo study in swine that demonstrated fibrillogenesis upon injecting subcutaneously along with long-term stability, no infection, and biocompatibility [42]. 

To determine if the liquid collagen was amendable to 3D printing, we utilized two different additive manufacturing printers. The first printer was a custom-built printer utilizing CNC milling motors along with a mounted syringe pump to create a 3D structure. The second printer was a commercial Cellink BioX bioprinter. Additionally, we investigated the extrusion pressure using the Cellink BioX bioprinter which provided a profile of extruded collagen fiber diameters. As shown in Figure 2 and Figure 3, both printers demonstrated that the liquid collagen was amendable to 3D printing. The liquid collagen was able to create stable 3D structures. Additionally, the liquid collagen could be printed into different patterns from a waffle design to hexagonal designs. Both printers were amendable to printing the collagen solution; however, the Cellink bioprinter provided more control over the extrusion pressures and temperatures and thus allowed for slightly more control over the resulting collagen fiber diameters. As shown in Figure 5, the diameter of the collagen increased from 400 μm to 800 μm when the extrusion pressure increased from 7 kPa to 13 kPa. There was a significant increase in the diameters of the printed fibers at 13 kPa. An earlier study by our group demonstrated that the injection force of the liquid collagen is quite low at 7 N, and it is assumed that the higher extrusion pressures result in a bolus of tangled pre-fibrillized collagen peptide chains being injected out of the printer head resulting in thicker fibers. 

Thermal stability analysis was also performed on various molar concentrations of the crosslinkers EDC/NHS and genipin to determine the potential stability of the 3D scaffolds. It was determined that both printing directly into a crosslinking solution and printing first in water and then crosslinking created more thermally stable scaffolds as compared to the uncrosslinked scaffolds. There was a concern that printing directly into a crosslinking solution may cause immediate crosslinking of carboxyl to amino groups, thereby disrupting the polymerization of the liquid collagen and leading to a less thermally stable structure. A destabilization (i.e., lower denaturation temperature) was observed by Municoy et al. when silver nanoparticles were added to the collagen ink prior to printing [44]. The authors inferred that AgNPs interfered with the self-assembling processes of collagen molecules. Our method of directly printing collagen into the crosslinking solution did not significantly interfere with the fibrillization; however, the water-first, two-step process created the most stable scaffold at higher exposure temperatures. This may be due to the collagen monomers being allowed to self-assemble naturally prior to chemical crosslinking. 

Genipin was also investigated as a potential crosslinker after printing the 3D scaffold. Genipin was able to create a more thermally stable scaffold relative to EDC/NHC crosslinking. However, genipin would be the preferred crosslinker because there are no known cytotoxic byproducts and would not require extensive washing of the resulting collagen scaffolds while EDC/NHS has cytotoxic urea as a byproduct and would require extensive washing to remove the byproduct. 

Both EDC/NHS and genipin were utilized to conjugate AuNPs to the 3D scaffolds to determine if the 3D-printed scaffolds were amendable to modifications. As shown in Figure 7 and Figure 8, AuNPs were visualized on the surface of the 3D scaffolds through SEM analysis. The SEM micrographs provide evidence that EDC/NHS may have induced more clumping of the AuNPs on the surface of the 3D scaffold while the use of genipin provided evidence of a more homogenous distribution of AuNPs over the 3D scaffold. The EDC/NHS is a fast reaction and thus may have resulted in a tendency of the AuNPs to be clumped quickly on the scaffold instead of more distribution as shown with the genipin. NAA analysis was also then conducted to determine the mass percent of AuNPs on the 3D scaffolds. Doubling the amount of AuNPs during crosslinking correlated to an approximate doubling in AuNPs conjugated to the 3D scaffolds in both cases of using EDC/NHS and genipin. Further work could be conducted to determine an approximate limit of conjugation with each concentration of EDC/NHS and genipin. Work with AuNPs has shown their potential as anti-inflammatory agents with a propensity toward cellular migration which are advantageous properties for potential in vivo scaffolds [31,45].

Cell studies were also performed to assess the biocompatibility and versatility of the 3D-printed collagen structures. We determined whether the collagen ink could be printed into a cell suspension. The advantage of this technique is that fibrillization would occur in the cell solution while also promoting cellular attachments. As shown in Figure 10, it was possible to fibrillize the collagen in the cell solution and enhance cellular attachments. The collagen was printed in linear strips, and the fibroblast cells are shown attaching to the printed collagen, even after repeated washing. We are one of the first groups to demonstrate direct 3D printing of liquid collagen into cell cultures to induce fibrillization and cellular attachment. 

Biocompatibility was also established via cell culture assays. A 3-day WST-1 cell viability analysis was performed on the 3D-printed collagen scaffolds that were crosslinked with genipin or EDC/NHS and conjugated with AuNPs. As noted in Figure 11A,B, it was apparent that the cells were viable on the 3D-printed scaffolds. However, the EDC/NHS crosslinked scaffolds with and without AuNPs demonstrated a significant reduction (*p* < 0.05) as compared to the control. On the other hand, the genipin-crosslinked scaffolds with and without AuNPs demonstrated a significant increase in cellularity at day 3 (*p* < 0.001) as compared to the control. Both the genipin-conjugated AuNP scaffolds and EDC/NHS-conjugated AuNP scaffolds demonstrated a reduction in the overall viability relative to the uncrosslinked scaffolds but still demonstrated high cellularity. The EDC/NHS scaffolds demonstrated a slightly reduced cellular viability relative to cells with no scaffold. It was concluded that genipin demonstrated a more cell-friendly crosslinker, which is in agreement with the published literature [46]. 

A 7-day WST-1 cell viability analysis was also performed only using genipin to crosslink AuNPs to the 3D scaffolds. At this time point, the cells with no scaffold were the most metabolically active. Interestingly, all samples crosslinked with genipin had an increased cellular viability relative to the uncrosslinked scaffold with 2× AuNP having the highest overall viability among groups with a scaffold. Comparing these results with the 3-day results, over a longer-term study, the genipin–AuNP scaffolds were favorable. 

As shown in Figure 12, agarose particles adhere to our 3D-printed scaffolds even after rigorous washing. This adherence most likely occurs due to the fibrillization that occurs once our liquid collagen interacts with the agarose bath. Printing in an agarose microparticle solution has the advantage of achieving enhanced reproducibility and 3D scaffold resolution [39], but there are drawbacks with the remnant agarose on the scaffolds. For example, there are previously published reports that demonstrated low attachment of cells to agarose [45,46]. The remnant agarose may prevent the necessary cell adhesion and migration to recapitulate new tissue. Additionally, washing may not be sufficient to remove the remnant agarose, and further more rigorous washing could damage the 3D-printed scaffolds. 

Figure 13 and Figure 14 display the results of seeding stromal cells onto the 3D-printed scaffolds. Determining cellular viability is one of the first steps for tissue-engineering various functional in vitro organs. The utilization of 3D-printing methodologies has given rise to new strategies to enhance tissue-engineered scaffolds, which involve either cell seeding the 3D-printed scaffolds or encasing cells in the ink for 3D printing [47]. Our initial work was performed with cell-seeding stromal cells to determine if the 3D-printed collagen scaffolds could remain structurally viable for long-term use. Other researchers have developed cell-laden inks; for example, Nulty et al. [48] 3D printed a fibrin-based hydrogel encased with human umbilical vein endothelial cells and human bone marrow stem/stromal cells to generate prevascularized tissues. Their 3D-printed scaffolds were able to support the establishment of a microvessel network. Shafiee et al. [49] 3D printed a biomimetically designed polycaprolactone and seeded the scaffolds with human gingival tissue multipotent mesenchymal stem/stromal cells for skin wound dressings. Their findings demonstrated that the 3D-printed mPCL scaffolds decreased wound contracture and improved skin regeneration. We initially utilized our uncrosslinked 3D collagen scaffolds that were seeded with stromal cells. As demonstrated in Figure 13, after 9 days of incubation, the stromal cells appeared to degrade the scaffold and preferred the bottom of the well plate. A revised scaffold was subsequently tested. The revised scaffold was crosslinked with genipin, and laminin was also added to help maintain the structural integrity and help the adherence of the stromal cells on the scaffold. After 16 days, the samples crosslinked with genipin remained structurally viable, and cells appeared to form a homogenous layer over the surface of the scaffold, as shown in Figure 14. These results indicated that cellular viability can be achieved and sustained in order to investigate various functional in vitro organs. 

## 5. Conclusions

We demonstrated the ability to 3D print liquid collagen into structural scaffolds. These collagen scaffolds were amendable to crosslinking as well as conjugating with gold nanoparticles. The scaffolds were characterized and demonstrated repeatability, stability, and cellular viability. The collagen ink has the possibility of developing into a plethora of different tissue-engineered structures. 

## 6. Patents

A U.S. patent was filed on 7 December 2021; Application No. 17/643,046.

## Data Availability

The data presented in this study are available on request from the corresponding author. The data are not publicly available due to privacy.
